# Exploring the infection strategy of *Colletotrichum fructicola* in pecan and two effectors Cf-ID1 and Cf-ID2 were characterized using unique molecular identifier-RNA sequencing technology

**DOI:** 10.3389/fpls.2025.1551342

**Published:** 2025-04-17

**Authors:** Long-Jiao Hu, Ji-Ping Xuan, Yang Li, Min Zhai, Guo-Ming Wang, Li-Na Deng, Zheng-Hai Mo

**Affiliations:** ^1^ Jiangsu Key Laboratory for the Research and Utilization of Plant Resources, Institute of Botany, Jiangsu Province and Chinese Academy of Sciences, Nanjing, Jiangsu, China; ^2^ Jiangsu Engineering Research Center for the Germplasm Innovation and Utilization of Pecan, Institute of Botany, Jiangsu Province and Chinese Academy of Sciences, Nanjing, Jiangsu, China; ^3^ School of Ocean and Biological Engineering, Yancheng Institute of Technology, Yancheng, Jiangsu, China

**Keywords:** *Colletotrichum fructicola*, *Carya illinoinensis*, UMI RNA-seq, effectors, immune response, infection suppression

## Abstract

The anthracnose disease caused by *Colletotrichum fructicola* has widely occurred in pecan (*Carya illinoinensis*) in China, seriously affecting its fruit yield and quality. However, the details of the infection strategy of *C. fructicola* remain to be elucidated. In this study, unique molecular identifier-RNA sequencing (UMI RNA-seq) was used to analyze differentially expressed genes (DEGs) of *C. fructicola* and candidate effectors were predicted. Two candidate effectors were identified during the early infection stages of *C. fructicola*. There were 6,822 DEGs at three infection timepoints (6, 24, and 36 h post-inoculation), and these genes were involved in spore germination, nutrient uptake, detoxification, secretion of toxic substances (such as effectors and toxins), inhibition of the host’s immune response, and protein post-translational modification, which participated in the pathogenic process of *C. fructicola*. Moreover, 191 candidate effectors were predicted and their expression trends were divided into five clusters. Two candidate effectors Cf-ID1 and Cf-ID2 were selected for functional validation, and they were demonstrated to trigger cell death and immune response in *Nicotiana benthamiana*. Cf-ID1 and Cf-ID2 are located in both cytoplasm and nucleus and could suppress the infection of *C. fructicola* by eliciting defense responses in *N. benthamiana*. This study provided valuable information for in-depth research on the pathogenesis of *C. fructicola*.

## Introduction

Pecan (*Carya illinoinensis* (Wangenh.) K. Koch), which belongs to the family Juglandaceae, genus *Carya*, is native to the United States and Mexico (Sparks, 2002). Due to the high economic value of its nuts and timber, pecan has been cultivated in many countries, including China, Australia, Argentina, and Peru (Bilharva et al., 2018). In China, this species is widely planted in Jiangsu and Anhui Provinces. With the continuous expansion of the planting area of pecan, disease severity has also increased, which has been a serious problem that has limited the development of the planting industry. Anthracnose caused by *Colletotrichum* spp. is one of the most serious diseases in pecan, which mainly infects the fruit and leaves (Chang et al., 2022; Meng et al., 2021). Among the numerous *Colletotrichum* spp. that infect pecan, the isolation rate of *Colletotrichum fructicola* is the highest, and it is a critical pathogen observed in pecan cultivation that impedes plant growth and overall fruit productivity (Meng, 2020).

*C. fructicola* has a broad host range and has been identified in over 50 plant species in the world, resulting in large economic losses (Liang et al., 2018). *C. fructicola* is a hemibiotroph with a multistage infection process (biotrophic and necrotrophic phases) (Liang et al., 2018). A previous study showed that factors contributing to *Colletotrichum* virulence, such as effectors and secondary metabolite (SM) biosynthetic enzymes, were generally expressed early in the infection process and functioned in host defense suppression or cellular activity manipulation (Baroncelli et al., 2016). At present, the widespread occurrence of apple anthracnose and *Camellia* anthracnose caused by *C. fructicola* has been further studied to explore the genes and pathways related to pathogenesis (Rockenbach et al., 2016; Liang et al., 2018). Chang et al. revealed the mechanisms of resistance to *Colletotrichum fioriniae* in two pecan varieties with different resistance levels through transcriptome sequencing (Chang et al., 2023). The potential pathways and genes involved in pecan resistance to *Pestalotiopsis microspore* were also explored (Chen et al., 2022). However, there are few studies on the interaction between *C. fructicola* and pecan thus far (Chang et al., 2022). With the increasingly serious occurrence of pecan anthracnose, exploring the pathogenesis of *C. fructicola* in pecan is greatly significant for the prevention and control of this disease.

In nature, plants are susceptible to various pathogens, including bacteria, fungi, oomycetes, viruses, and parasitic nematodes. To defend against pathogens, plants have developed an immune system that recognizes microbial invaders and activates defense mechanisms to prevent their proliferation. At the same time, pathogens secreted effectors to manipulate plant immunity pathways or create a favorable environment to facilitate pathogen colonization. The latest definition of an effector is: a pathogen-secreted molecule that functions to induce non-self-phenotypes in other organisms or the environment (Ai et al., 2024). Numerous studies have shown that the consequences of plant-pathogen interactions are largely determined by effectors (Jones and Dangl, 2006; Wang et al., 2018).

RNA-sequencing technology has been widely adopted to investigate host responses during pathogen infection (Foster et al., 2015; Li et al., 2019) or to identify the associated molecular cellular pathways and virulence factors (such as effectors) of pathogens secreted during the infection process (Zuluaga et al., 2016; Hu et al., 2021). Although RNA-seq is a powerful tool, sequence-dependent bias and the inaccuracy of PCR amplification limit its further applications (Chang et al., 2023). To solve this problem, in UMI RNA-seq, each cDNA molecule is labeled with a unique molecular identifier (UMI) before library construction (Smith et al., 2017).

In this study, UMI RNA-seq was applied to explore the infection strategy of *C. fructicola* in pecan during the early stages. Particular emphasis was placed on the identification of differentially expressed genes (DEGs), Gene Ontology (GO) enrichment, and Kyoto Encyclopedia of Genes and Genomes (KEGG) pathway analysis of DEGs of *C. fructicola*. The candidate effectors of *C. fructicola* were predicted. To focus on the relationship between the candidate effectors and their corresponding enriched functions, the expression trends of the candidate effectors were divided. Then, two candidate effectors were selected for functional validation and were characterized via the transient expression in *Nicotiana benthamiana*. The abilities of the two effectors to trigger plant immunity and suppress *C. fructicola* infection, as well as their subcellular localization, were examined.

## Materials and methods

### Biological material

The *C. fructicola* isolate CZ102 was originally isolated from leaf spot lesions on pecan in Chuzhou City, Anhui Province, China. The isolate CZ102 was subcultured on potato dextrose agar (PDA) at 25°C and preserved using filter paper at -20°C in the Fungal Laboratory, Fruit Tree Research Center, Institute of Botany, Jiangsu Province and Chinese Academy of Sciences. Seedling progenies derived from an open-pollinated cultivar (‘Pawnee’) were used as plant materials. The seedlings were cultivated as previously described (Mo et al., 2023).

### Sample preparation

In a preliminary study, the appressorium and primary hyphae (infection structures) of *C. fructicola* began to form on onion epidermis at 6 h and 24 h, respectively (**Supplementary Figure S1**). A previous study found that *C. fructicola* formed appressorium at 8 h after inoculation with cellophane, and formed a large number of primary hyphae at 36 h after inoculation (Liang et al., 2018). Thus, three infection timepoints [6, 24, and 36 h post-inoculation (hpi)] were finally determined for this study. Specifically, the *C. fructicola* isolate CZ102 was cultured in a complete medium (CM) to produce conidia as previously described. The CM composition per liter is: sucrose 20 g, yeast extract 6 g, and casamino acid 6 g (Huang et al., 2017). Conidial suspensions were adjusted to 1×10^6^ spores/mL and sprayed on healthy leaves of 3-month-old pecan seedlings. The inoculated seedlings were kept in a moist chamber at 25°C (80% relative humidity and 12 h light and 12 h dark cycles). The leaves of pecan seedlings were collected after being inoculated with conidial suspensions for 6 h (CP_6 h, group B), 24 h (CP_24 h, group C), and 36 h (CP_36 h, group D). Purified conidia of *C. fructicola* were used as a control (group A). For each group, three independent samples were immediately frozen in liquid nitrogen and stored at -80°C for subsequent RNA extraction and sequencing.

### RNA isolation, library preparation, and sequencing

The UMI RNA-seq experiment, the high throughput sequencing, and basic RNA-Seq data analysis were conducted by Seqhealth Technology Co., LTD (Wuhan, China).

The total RNA of all samples was extracted using TRIzol Reagent (Invitrogen, cat. No. 15596026)following the methods of Chomczynski et al (Chomczynski and Sacchi, 1987). DNA digestion was carried out using DNase I. RNA quality was determined by examining A260/A280 with a NanoDrop One C spectrophotometer (Thermo Fisher Scientifific, Inc.). The RNA integrity of 12 samples was confirmed using a 5300 Fragment Analyzer system (Agilent). Qualified RNAs of total samples were finally quantified by Qubit 3.0 with a Qubit RNA broad-range assay kit (Life Technologies, Q10210).

Two μg total RNAs were used for stranded RNA sequencing library preparation using a KC-DigitalTM Stranded mRNA Library Prep Kit (Catalog NO. DR08502, Wuhan Seqhealth Co., Ltd. China) following the manufacturer’s instructions. The kit eliminates duplication bias in PCR and sequencing steps by using a UMI of eight random bases to label the pre-amplified cDNA molecules. The library products corresponding to 200-500 bps were enriched, quantified, and finally sequenced on a DNBSEQ-T7 sequencer (MGI Tech Co., Ltd. China) with the PE150 model.

### RNA-seq data analysis

Raw sequencing data of the four groups (A, B, C, and D) were first filtered using fastp (version 0.23.0). Then, the low-quality reads were discarded and the reads contaminated with adaptor sequences were trimmed. The clean reads were further treated with in-house scripts to eliminate duplication bias introduced in library preparation and sequencing. In brief, the clean reads were first clustered according to the UMI sequences, in which reads with the same UMI sequences were grouped into the same cluster. Reads in the same cluster were compared to each other by pairwise alignment, and then reads with sequence identity over 95% were extracted to a new sub-cluster. After all sub-clusters were generated, multiple sequence alignment was performed to get one consensus sequence for each sub-cluster. After these steps, any errors and biases introduced by PCR amplification or sequencing were eliminated.

The de-duplicated consensus sequences were used for standard RNA-seq analysis. They were mapped to the reference genome of *C. fructicola* from the National Center for Biotechnology Information (https://www.ncbi.nlm.nih.gov/genome/57524?genome_assembly_id=750361) using STAR software (version 2.5.3a) with default parameters. The reads mapped to the exon regions of each gene were counted by featureCounts (Subread-1.5.1; Bioconductor) and then Fragments Per Kilobase of transcript per Million mapped reads (FPKM) was calculated. The DEGs between groups were identified using the edgeR package (version 3.12.1). An adjusted P-value < 0.05 and absolute log_2_ (fold change) ≥ 1 were used to judge the statistical significance of gene expression differences. Among the DEGs, upregulated genes of *C. fructicola* had a higher expression at least at one infection timepoint (B, C, or D) than at the conidia stage (A), and downregulated genes of *C. fructicola* had a lower expression at all the infection timepoints than at the conidia stage.

### GO enrichment analysis of DEGs in *C. fructicola*

The GO enrichment analysis of *C. fructicola* upregulated genes from the above transcriptomic data (CP_6 h, CP_24 h, CP_36 h) was implemented by the GOseq R package, in which gene length bias was corrected (Young et al., 2014). Furthermore, DEGs were selected for biological process, cellular component, and molecular function analysis using the Rich factor as the screening indicator. GO terms with an adjusted P-value (i.e., false discovery rate, FDR) < 0.05 were considered significantly enriched.

### KEGG enrichment analysis of DEGs in *C. fructicola*

The KEGG is a database resource for understanding the high-level functions and utilities of biological systems (http://www.genome.jp/kegg/) (Kanehisa et al., 2016). KOBAS software (version: 2.1.1) was used to determine the statistical enrichment (with adjusted P value < 0.05) of DEGs in KEGG pathways. Furthermore, to improve the annotation, a BLAST search was performed against the SWISS-Prot database.

### Real-time quantitative PCR analysis

To validate the accuracy of UMI RNA-seq data, real-time quantitative polymerase chain reaction (RT-qPCR) assays were carried out in a CFX Opus 96 real-time PCR system (Bio-Rad, USA) with ChamQ SYBR qPCR Master Mix (Low ROX Premixed) (Vazyme, China) according to the manufacturer’s instructions. The relative expression levels of seven DEGs in *C. fructicola* were detected. PCR amplification was performed under the following conditions: 95°C for 30 s, followed by 40 cycles of 95°C for 10 s and 60°C for 30 s. The alpha-tubulin (*TUB*) gene of *C. fructicola* was chosen as a constitutively expressed endogenous control gene (Liang et al., 2018; Shang et al., 2020). The relative quantifications were calculated based on the 2^-ΔΔCt^ method. Primer sequences are provided in **Supplementary Table S1**.

### Candidate effectors prediction and analysis

Potential effectors were screened according to several criteria (Stergiopoulos and De Wit, 2009). Briefly, they should be upregulated genes during the pathogen-host interaction stage. At the same time, there should be the presence of an N-terminal signal peptide and the absence of a transmembrane domain in the amino acid sequences of potential effectors. Moreover, the sequence length of potential effectors should be ≤ 300 amino acids (aa). Candidate effectors screened according to the above requirements were predicted to further distinguish between apoplastic and cytoplasmic effectors using EffectorP 3.0 (https://effectorp.csiro.au/) (Sperschneider and Dodds, 2022). The signal peptide and the transmembrane domain were predicted by SignalP5.0 (http:///services.healthtech.dtu.dk/services/SignalP-5.0/) (Almagro Armenteros et al., 2019) and TMHMM 2.0 (http://www.cbs.dtu.dk/services/TMHMM-2.0/) (Sonnhammer et al., 1998), respectively. A BLASTP search of all candidate effectors against the Non-Redundant (NR) database was conducted to predict their functions. The expression trends of candidate effectors were clustered using the R package Mfuzz and the membership degree represented the importance of the gene in the cluster to a certain extent.

### Plasmid construction

Referring to the previously used method (Hu et al., 2019), three candidate effectors were cloned from the cDNA of *C. fructicola* using the specific primers listed in **Supplementary Table S1**. Subsequently, purified PCR products were ligated into the pBINGFP vector using the *ApexHF* HS DNA Polymerase FS (Accurate Biotechnology (Hunan) Co., LTD, Changsha, China) after confirmation by sequencing.

### Transient expression assays in *Nicotiana benthamiana*

The constructed plasmids of the candidate effectors were inserted into *Agrobacterium tumefaciens* GV3101 by electroporation. The culture conditions of *A. tumefaciens* GV3101, carrying candidate effectors, components of washing buffer, and a final optical density (OD) of 600 nm of *A. tumefaciens* suspensions, were the same as those in our previous study (Hu et al., 2020). For agroinfiltration assays, the *A. tumefaciens* suspensions were infiltrated into the leaves of *N. benthamiana* using a needleless syringe. Symptoms of *N. benthamiana* were observed visually 7 days after infiltration. Both the empty vector pBINGFP and a candidate effector Cf-1 (Gene ID: CGMCC3_g6914), which has been previously validated to lack necrosis-inducing ability in *N. benthamiana* (unpublished data), were used as negative controls.

To test the suppression of *C. fructicola* infection by Cf-ID1 and Cf-ID2, pBINGFP-Cf-ID1, pBINGFP-Cf-ID2, and pBINGFP were infiltrated into the leaves of *N. benthamiana*. The leaves were detached at 36 h post-infiltration, and then inoculated on the infiltrated regions with 4 mm hyphae blocks of *C. fructicola*. Under dark conditions at 72 h post-infiltration, the leaves were illuminated with a handheld UV lamp (LUYOR-3410UV, Shanghai Luyor Biotechnology Co., Ltd, Shanghai, China) for clear observation of the lesion dimensions. At the same time, the disease lesions were photographed. For Cf-ID1 and Cf-ID2, the experiment was respectively performed three times, and three different plants with two inoculated leaves were used for each assay.

To determine whether candidate effectors Cf-ID1 and Cf-ID2 can trigger a PAMP-triggered immunity (PTI) response, the relative expressions of three PTI marker genes (*NbAcre31*, *NbPTI5*, and *NbCyp71D20*) (Nie et al., 2018) were detected at 3 h post-infiltration and 12 h post-infiltration with *A. tumefaciens* GV3101 carrying pBINGFP-Cf-ID1 and pBINGFP-Cf-ID2 in *N. benthamiana*, respectively. *N. benthamiana* infiltrated with pBINGFP was the control. NbEF1α of *N. benthamiana* was used as the constitutively expressed endogenous control gene (Ma et al., 2015). The amplification conditions of RT-qPCR were consistent with those mentioned above. The relative quantifications were calculated based on the 2^-ΔΔCt^ method. The assay was performed three times. Primer sequences are provided in **Supplementary Table S1**.

### Subcellular localization in *N. benthamiana*

The *N. benthamiana* leaves were agroinfiltrated with pBINGFP-Cf-ID1 and pBINGFP-Cf-ID2, and the P19 silencing suppressor in a 2:1 ratio at a final OD600 = 0.5 for each construct, respectively. The *N. benthamiana* leaves agroinfiltrated with empty vector pBINGFP were used as controls. Furthermore, 36 h after agroinfiltration, patches of *N. benthamiana* leaves were cut and mounted in water and analyzed using an LSM900 laser scanning microscope (Zeiss, Germany).

## Results

### Pathogenicity of *Colletotrichum fructicola* to pecan

Leaves of healthy 3-month-old pecan seedlings were sprayed with conidial suspensions of *C. fructicola*. Black spot symptoms appeared on the leaves of pecan 3 days after inoculation. We re-isolated, purified, and sequenced the pathogen from the spots, and the results showed that they were consistent with the initially inoculated *C. fructicola*. Therefore, the pathogenicity of *C. fructicola* to pecan was confirmed. The conidia morphology and susceptible pecan are shown in **Supplementary Figure S2**.

### Summary data of the transcriptome for *Colletotrichum fructicola*

In total, we generated 1,064,785,506 raw reads and 886,242,942 clean reads from 12 samples by UMI RNA-seq. Sample A (*C. fructicola* conidia) produced no less than 7 Gb of data, and Samples B, C, and D (mixed samples of *C. fructicola* conidia and plant tissue) produced no less than 13 Gb of data. The percentage of Q30 of all samples reached more than 89.85%, and the GC content was 46.83%–54.58% (**Supplementary Table S2**). In the B, C, and D groups, a total of 678 million reads were obtained, of which approximately 1.36% were aligned to the *C. fructicola* genomes. For the A group, there were 112 million reads in three samples, and approximately 96.62% of the reads were aligned to the *C. fructicola* genomes. The complete list, annotations, and FPKM of all transcripts are shown in **Supplementary Table S3**.

### Differentially expressed genes of *Colletotrichum fructicola*

**Figure 1:**
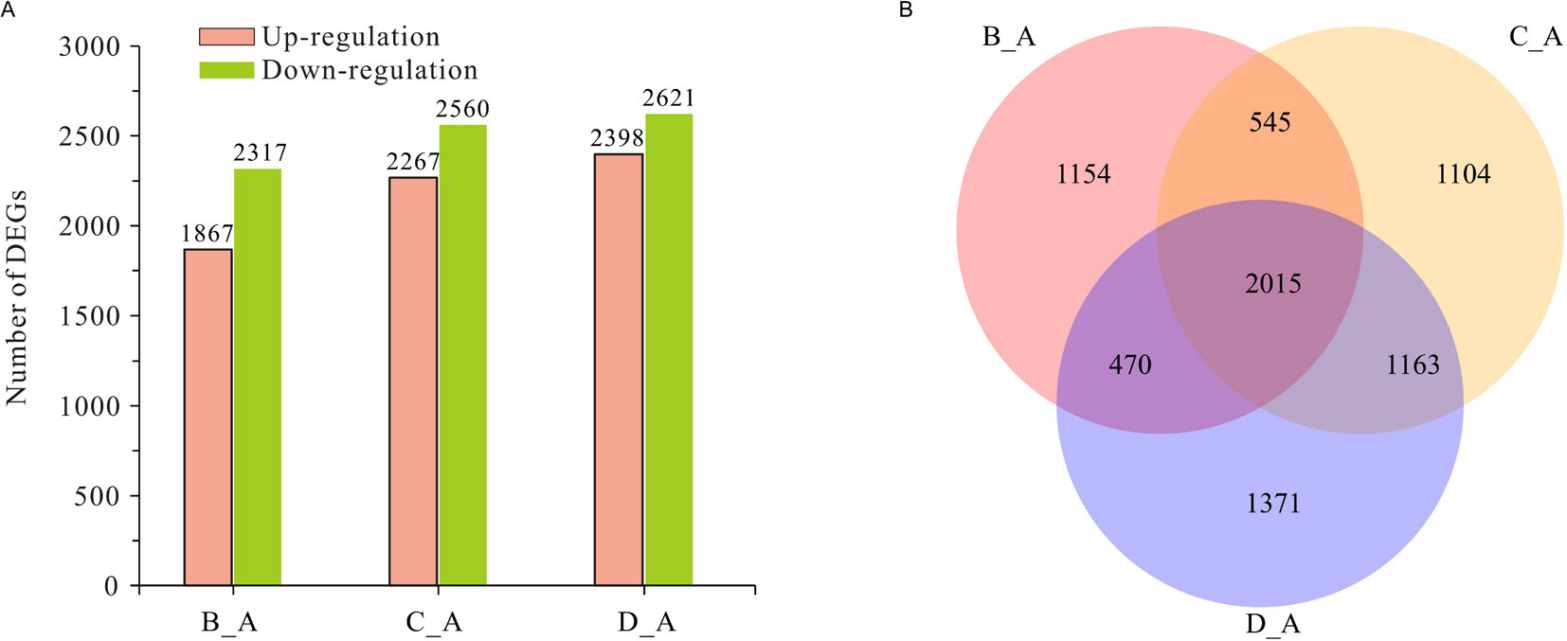
The analysis of differentially expressed genes (DEGs) in *Colletotrichum fructicola* during the early stages of pecan infection. **(A)** The numbers of DEGs of *C. fructicola*. **(B)** The Venn diagram analysis of DEGs of *C. fructicola*. **A** *C. fructicola* conidia. **B** 6 h post-inoculation (hpi). **C** 24 hpi. **D** 36 hpi.

In this study, gene expression levels were estimated via the FPKM method. The genes with a P-value < 0.05 and |log2 (fold change) | ≥1 were designated as DEGs between the early stages of infection (6, 12 and 24 h post-infection) and the conidia stage (0 h). Finally, there were 7,822 DEGs of *C. fructicola* at the three infection points, and the number of DEGs at 6 h, 24 h, and 36 h was 4,184 (1,867 upregulated genes and 2,317 downregulated genes), 4,827 (2,267 upregulated genes and 2,560 downregulated genes), and 5,019 (2,398 upregulated genes and 2,621 downregulated genes), respectively (**Figure 1A**). The list and annotation information and heatmap of all DEGs in *C. fructicola* are shown in **Supplementary Table S4** and **Supplementary Figure S3**, respectively. According to the Venn diagram analysis, 3,648 genes were DEGs at two infection timepoints, and 2,015 genes were DEGs at all three infection periods (**Figure 1B**). The Venn diagram analysis of 4,284 upregulated genes in the *C. fructicola* transcriptome during the early stages of infection is shown in **Supplementary Figure S4**.

### GO functional annotation and KEGG functional enrichment analysis of DEGs of *C. fructicola*

**Figure 2:**
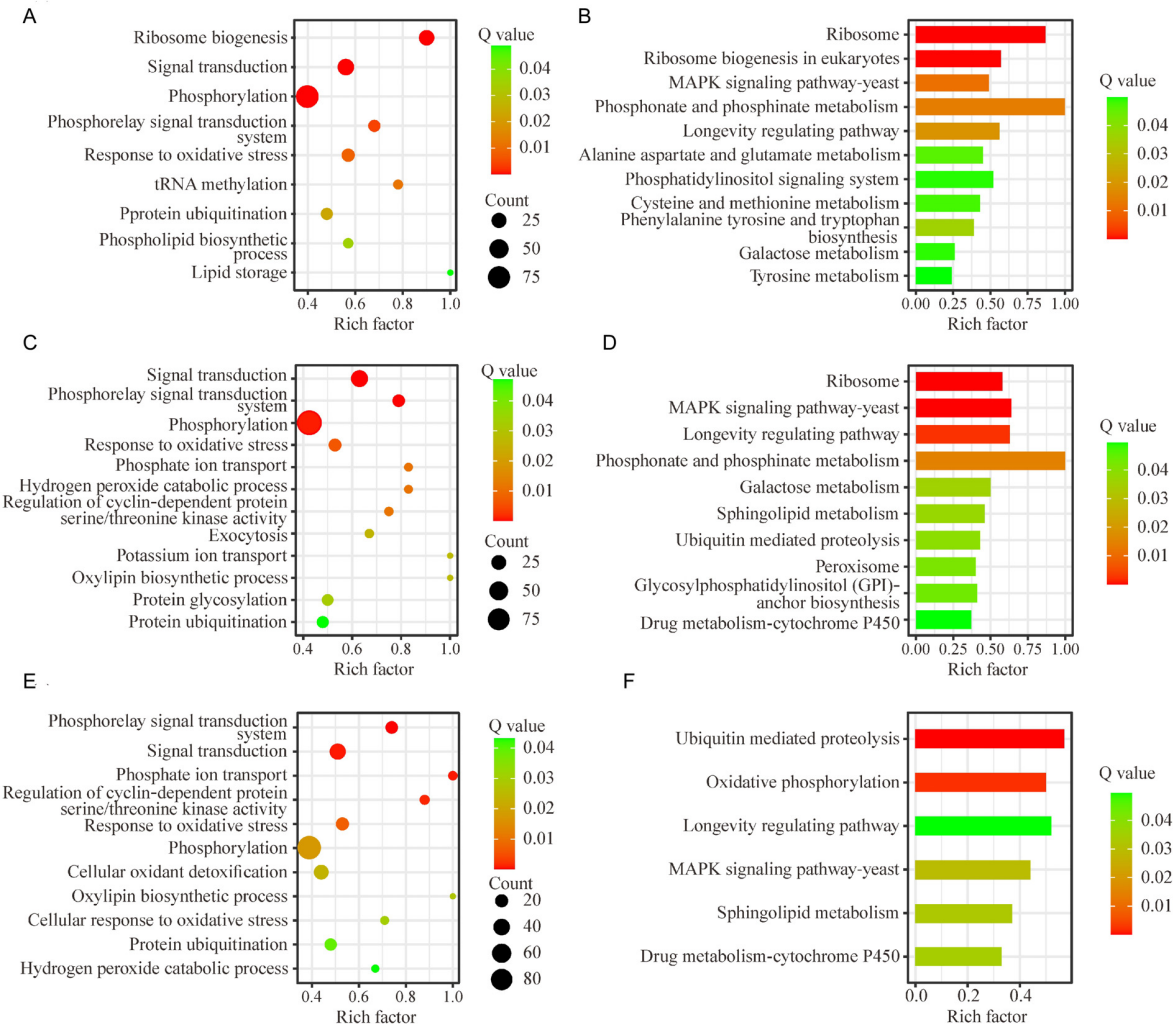
GO functional annotation and KEGG functional enrichment analysis of differentially expressed genes (DEGs) in *Colletotrichum fructicola* during the early stages of pecan infection. **(A, C, E)** Representative GO terms of DEGs in the *C. fructicola* transcriptomes at 6 h, 24 h, and 36 h post-infection, respectively. **(B, D, F)** Representative KEGG pathways of DEGs in the *C. fructicola* transcriptomes at 6 h, 24 h, and 36 h post-infection, respectively. **A**: *C. fructicola* conidia. **B**: 6 h post-inoculation (hpi). **C**: 24 hpi. **D**: 36 hpi.

In the *C. fructicola* transcriptomes at 6 hpi, the DEGs of *C. fructicola* showed significant enrichment in terms of post-transcriptional modifications, such as phosphorylation, tRNA methylation, and protein ubiquitination. Ribosome biogenesis, phosphorelay signal transduction system, signal transduction and response to oxidative stress, lipid storage, and phospholipid biosynthetic process were significantly enriched (**Figure 2A**). Moreover, the longevity regulating pathway, phosphonate and phosphinate metabolism, alanine aspartate and glutamate metabolism, phosphatidylinositol signaling system, cysteine and methionine metabolism, galactose metabolism, phenylalanine tyrosine and tryptophan biosynthesis, and MAPK signaling pathway were also significantly enriched (**Figure 2B**).

In the *C. fructicola* transcriptomes at 24 hpi, the hydrogen peroxide catabolic process, oxylipin biosynthetic process, and response to oxidative stress were significantly enriched. Furthermore, exocytosis, potassium ion transport, phosphate ion transport, protein ubiquitination, and glycosylation were also significantly enriched (**Figure 2C**), which have been proven to be involved in nutrient intake, adaptation to new environment, and inhibition of the host’s immune response for successful infection and reproduction (Lamarche et al., 2008; Chen et al., 2014, 2017; Qiu and Luo, 2017). At the same time, pathways associated with sphingolipid metabolism, ubiquitin-mediated proteolysis, peroxisome, and glycosylphosphatidylinositol (GPI)-anchor biosynthesis, and cytochrome P450 were significantly enriched (**Figure 2D**).

In the *C. fructicola* transcriptomes at 36 hpi, the oxylipin biosynthetic process, response to oxidative stress, cellular oxidant detoxification, cellular response to oxidative stress, and hydrogen peroxide catabolic process were significantly enriched (**Figure 2E**). Metabolic pathways, such as ubiquitin-mediated proteolysis, oxidative phosphorylation, sphingolipid metabolism, and cytochrome P450, were significantly enriched (**Figure 2F**).

Notably, terms related to toxic response, survival and reproduction, and immune recognition, such as the phosphorelay signal transduction system, signal transduction and response to oxidative stress, phosphorylation, and protein ubiquitination, were significantly enriched at these three timepoints (**Figures 2A, C, E**). Moreover, the MAPK signaling pathway and longevity regulating pathway were significantly enriched at these three timepoints (**Figures 2B, D, F**). These results suggested that during the early stage of infection, *C. fructicola* might not only be forced to adapt to the host environment under oxidative stress but also actively initiates aggression (such as secreting toxic substances and evading host immune recognition).

### Confirmation of the accuracy of the transcriptome data by qRT-PCR

**Figure 3:**
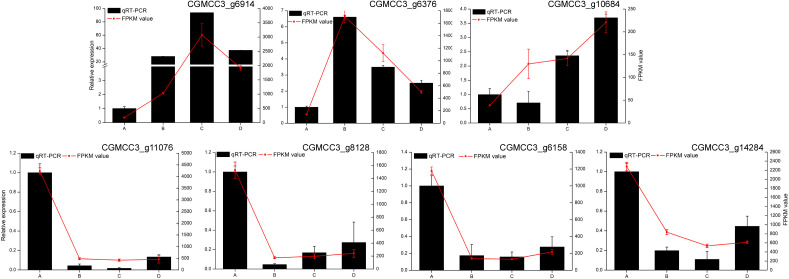
Validation of *Colletotrichum fructicola* transcriptome data. Bars: relative expression levels of differentially expressed genes (DEGs) in *C. fructicola* by real-time quantitative (RT-qPCR); red line: FPKM in the *C. fructicola* transcriptomes. Data are the means, and the error bars represent ± SD from three biological replicates. Different letters on top of the bars indicate statistically significant differences (p < 0.05, t-test) as measured by Duncan’s multiple range test.

To validate the accuracy of UMI RNA-seq, the transcript levels of seven DEGs from *C. fructicola* were determined by RT-qPCR. The trends obtained by RT-qPCR were highly consistent with the RNA-Seq results (**Figure 3**). This result indicated that the UMI RNA-seq data was reliable, and can be used for subsequent analysis.

### Screen and functional annotation of candidate effectors of *C. fructicola*

Based on the screening criteria (Stergiopoulos and De Wit, 2009) for effectors, the amino acid sequences encoded by 666 upregulated genes contained signaling peptides, and 550 of them encoded amino acids without transmembrane domains. Thus, 550 genes were preliminarily screened from 4,284 upregulated genes of *C. fructicola* during the early stages of infection. Among them, there were 191 candidate effectors whose sequence length was less than 300 aa. The list of these candidate effectors is provided in **Supplementary Table S5**. Among them, 126 candidate effectors were predicted to be apoplastic or cytoplasmic effectors by EffectorP 3.0 (**Supplementary Table S6**). The 191 candidate effectors included several categories, such as pioneer genes without any annotation, putative toxins (including necrosis-inducing proteins, NLPs), small secreted proteins, glycohydrolase, cell wall degrading enzymes (pectate lyase), and surface-binding proteins (**Supplementary Figure S5**).

**Figure 4:**
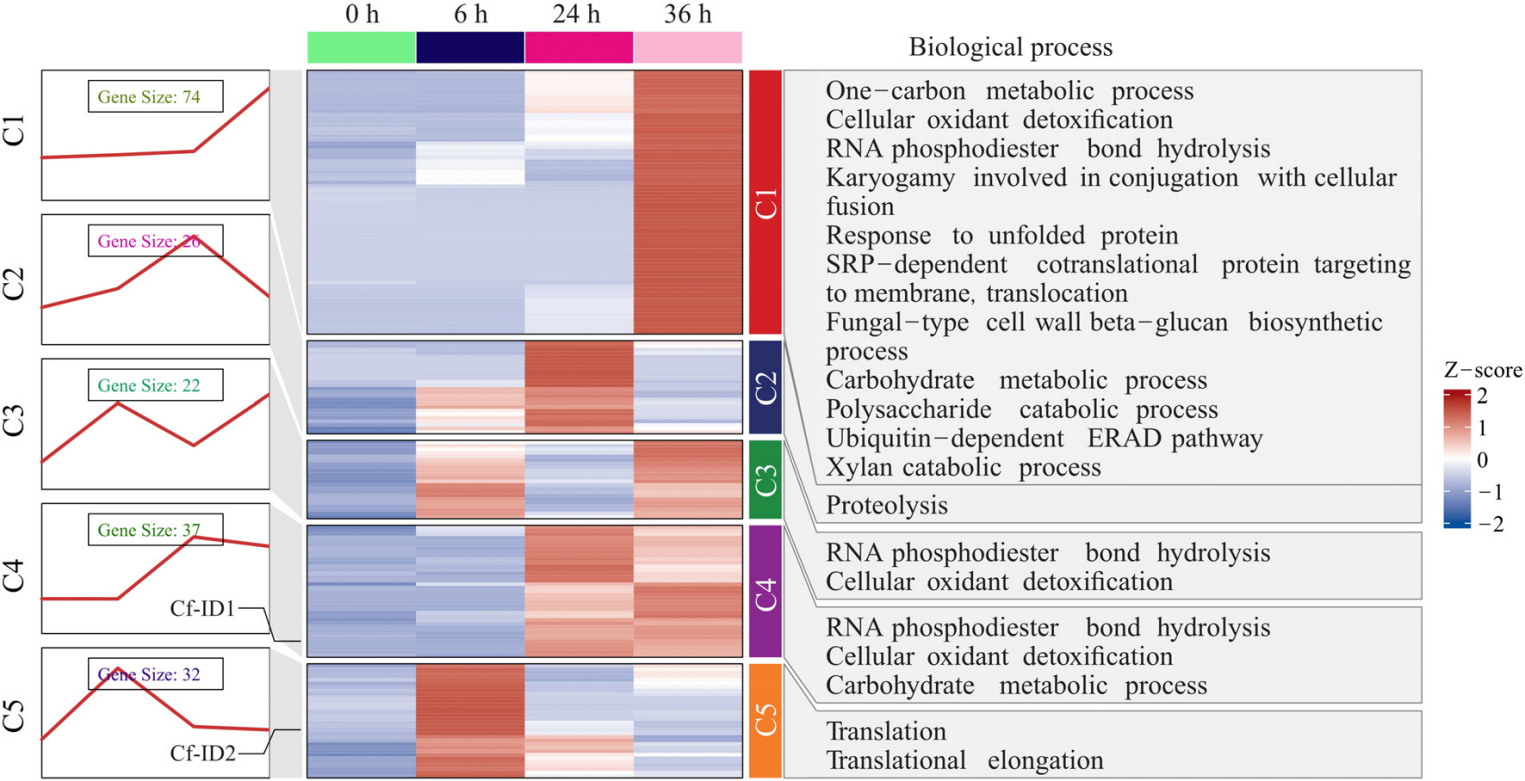
The expression trend analysis of 191 candidate effectors in *Colletotrichum fructicola*. Expression trends of 191 candidate effectors in *C. fructicola* were categorized into five clusters and biological processes with significant enrichment of candidate effectors were shown for each cluster.

Moreover, the expression trends of 191 candidate effectors were further categorized to elucidate their potential functions. The result showed that they were divided into five clusters and the corresponding enriched biological processes of candidate effectors under these expression trends were also shown (**Figure 4** and **Supplementary Table S7**). In the C1 and C3 clusters, the candidate effectors demonstrated maximal expression abundance at 36 hpi. In the C2 and C4 clusters, the candidate effectors demonstrated maximal expression abundance at 24 hpi. In the C5 cluster, these candidate effectors demonstrated maximal expression abundance at 6 hpi. GO enrichment analysis further indicated these candidate effectors were significantly enriched in the pathogenicity-related terms of cellular oxidant detoxification, RNA phosphodiester bond hydrolysis, translational elongation, xylan catabolic process, proteolysis, and ubiquitin-dependent ERAD pathway. It showed that *C. fructicola* secreted different effectors to participate in the pathogenic process at different timepoints and effectors were involved in various biological processes of *C. fructicola* in the process of infecting pecan.

### Cf-ID1 and Cf-ID2 induce cell death and immune responses and suppress infection in *Nicotiana benthamiana*

Based on the expression trends and corresponding GO annotation information, the candidate effectors in the C4 and C5 clusters were prioritized for further analysis. Then, after a comprehensive analysis of FPKM and the membership in the corresponding clusters (**Supplementary Table S7**), two candidate effectors (Gene ID: CGMCC3_g883 and CGMCC3_g5994) and one candidate effector (Gene ID: CGMCC3_g12505) met the requirements (mean FPKM >100 and membership >0.75) in the C4 and C5 clusters, respectively. Finally, the one unannotated candidate effector, Cf-ID1 (Gene ID: CGMCC3_g883), and Cf-ID2 (Gene ID: CGMCC3_g12505) were selected for functional validation in *N. benthamiana*. Their annotation information is provided in **Supplementary Table S8**.

**Figure 5:**
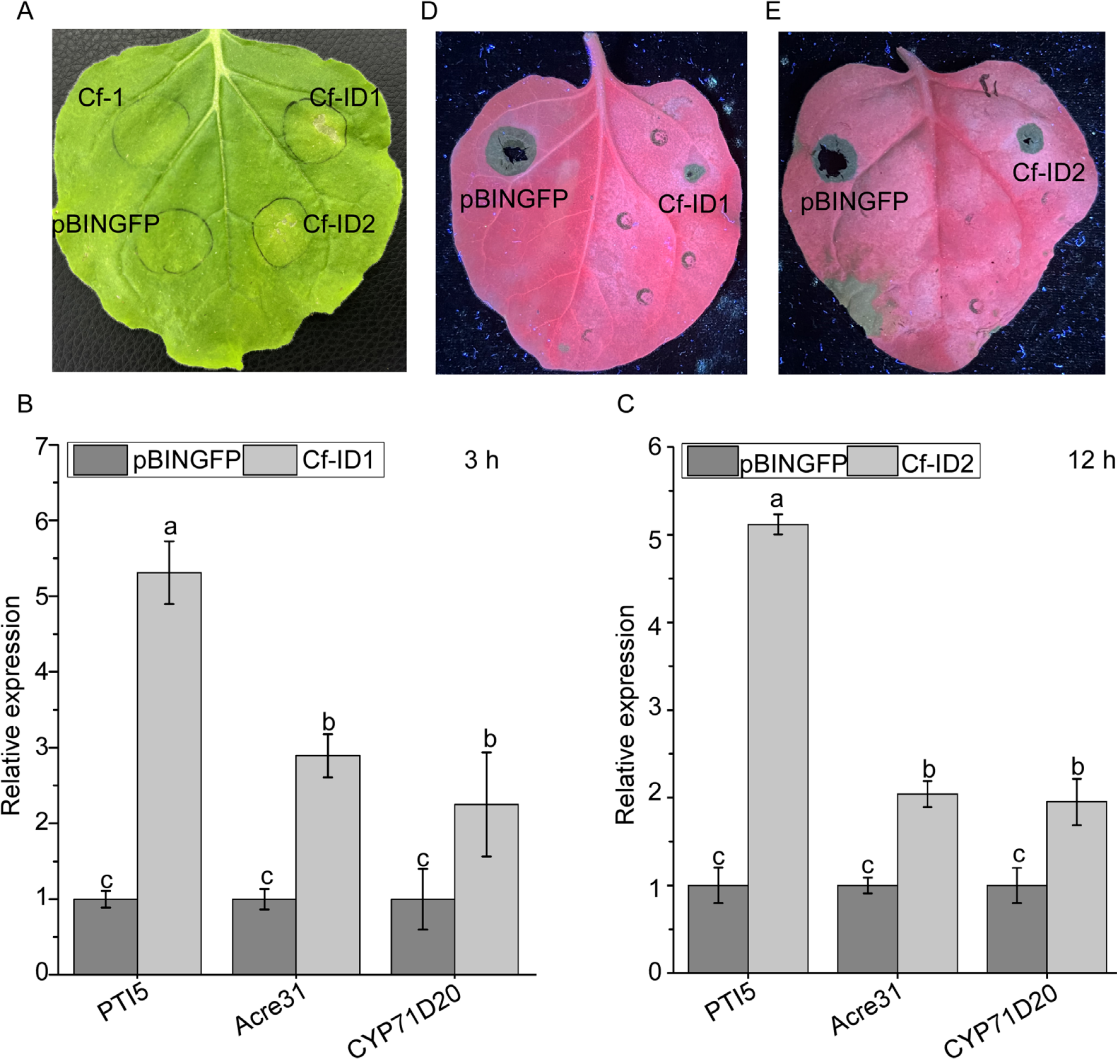
Cf-ID1 and Cf-ID2 could induce cell death and immunity response in *Nicotiana benthamiana.* **(A)** Representative *N. benthamian*a leaves at 7 days after agroinfiltration with *Agrobacterium* sp. strain GV3101 carrying pBINGFP-Cf-ID1 and pBINGFP-Cf-ID2*. N. benthamiana* infiltrated with GV3101 carrying the empty vector pBINGFP or the candidate effector Cf-1 (Gene ID: CGMCC3_g6914) were used as negative controls. Cf-1 has been previously validated to lack necrosis-inducing ability in *N. benthamiana* (unpublished data). These infiltration assays were separately performed three times, and three different plants with three inoculated leaves were used in each assay. **(B)** Transcriptional upregulation of three *N. benthamiana* PAMP-triggered immunity (PTI) marker genes infiltrated with pBINGFP-Cf-ID1 for 3 h **(C)** Transcriptional upregulation of *N. benthamiana* PTI marker genes infiltrated with pBINGFP-Cf-ID2 for 12 h *N. benthamiana* infiltrated with *Agrobacterium* sp. strain GV3101 carrying empty vector pBINGFP for 3 h and 12 h were used as controls. The experiment was repeated three times with similar results. Data are the means, and the error bars represent ± SD from three biological replicates. Different letters on top of the bars indicate statistically significant differences (p < 0.05, t-test) as measured by Duncan’s multiple range test. **(D, E)** Defense response induced by pretreatment with pBINGFP-Cf-ID1 and pBINGFP-Cf-ID2 and then inoculated 36 h later with *C. fructicola* in *N. benthamiana*. The left area of the leaves was separately infiltrated with GV3101 carrying pBINGFP-Cf-ID1 and pBINGFP-Cf-ID2, and the right area of the leaves was infiltrated with GV3101 carrying empty pBINGFP. The experiment was replicated three times with three plants per biological replicate and two leaves analyzed per plant. Disease lesions were assessed under UV light at 72 h post-infiltration.

The transient expression assay result showed that Cf-ID1 and Cf-ID2 induce cell death of *N. benthamiana* (**Figure 5A**). The result of RT-qPCR analysis showed that the relative expressions of *NbAcre31*, *NbPTI5*, and *NbCyp71D20* were significantly increased when infiltrated with pBINGFP-Cf-ID1 and pBINGFP-Cf-ID2 for 3 h and 12 h, respectively (**Figures 5B, C**).

In addition, to further assess whether Cf-ID1 and Cf-ID2 can suppress infection, *N. benthamiana* leaves were treated separately with pBINGFP-Cf-ID1 and pBINGFP-Cf-ID2 or with empty vector pBINGFP, and then 36 h later were inoculated with hyphae blocks of *C. fructicola*. At 72 h post-infiltration, the lesions that had been pretreated with the control were significantly larger than those of the pBINGFP-Cf-ID1 and pBINGFP-Cf-ID2-pretreated leaves in *N. benthamiana* (**Figures 5D, E**).

### Cf-ID1 and Cf-ID2 are located in both the cytoplasm and nucleus in *N. benthamiana*

**Figure 6:**
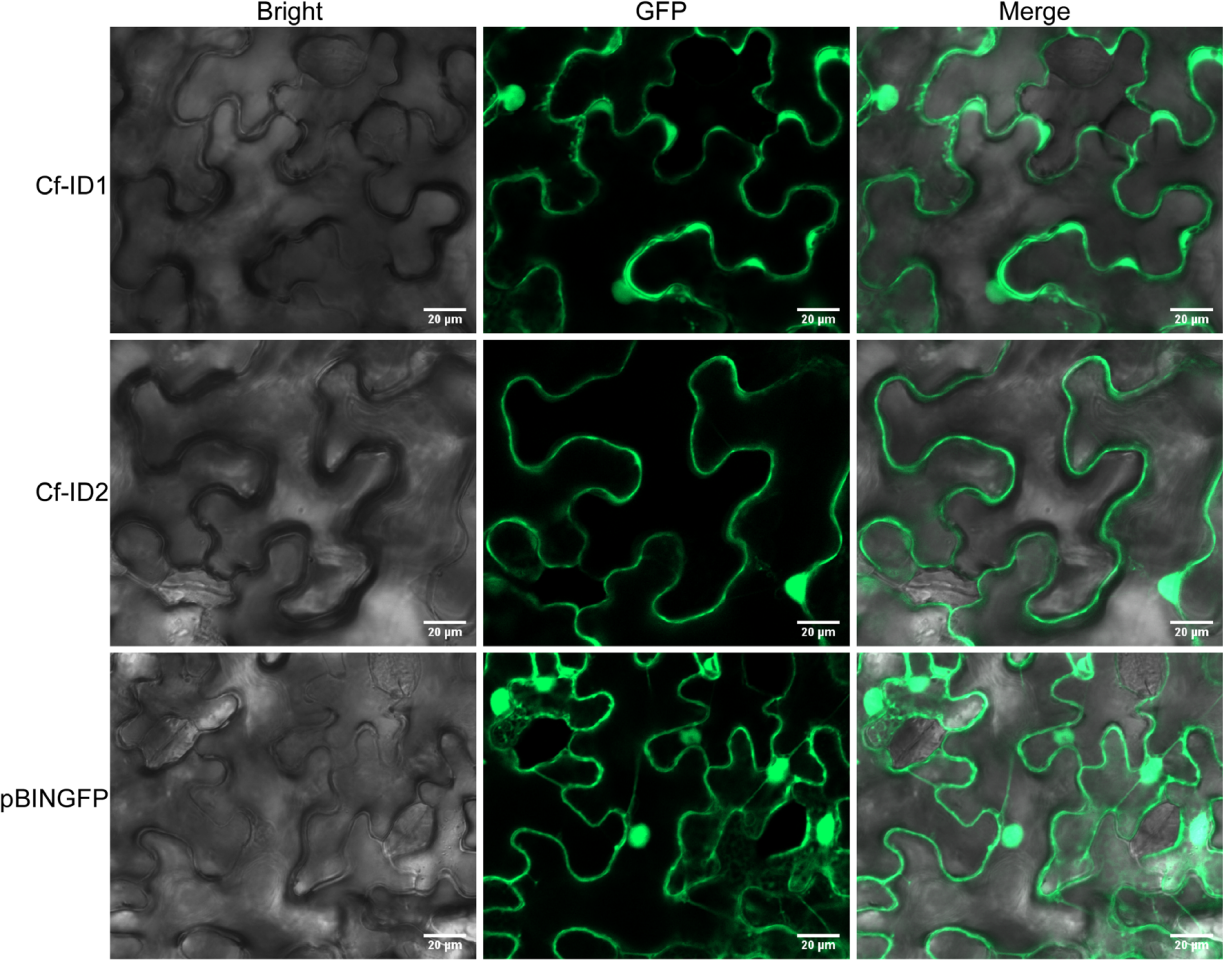
The subcellular localization of Cf-ID1 and Cf-ID2 in *Nicotiana benthamiana*. The subcellular localization of Cf-ID1 and Cf-ID2 were determined by transient expression of green fluorescent protein (GFP)-tagged proteins in *N. benthamiana* leaves. Confocal microscopy images were taken at 36 h post-infiltration.

According to the EffectorP 3.0 software prediction, the possibilities of these two candidate effectors being cytoplasmic effectors were 70.1% and 88%, respectively. To determine the subcellular localization of Cf-ID1 and Cf-ID2, we expressed C-terminal GFP-tagged Cf-ID1 and Cf-ID2 (without a signal peptide). Upon the expression of pBINGFP-Cf-ID1 and pBINGFP-Cf-ID2 in *N. benthamiana* leaves, GFP-derived fluorescence was detected both in the nucleus and cytoplasm (**Figure 6**). The result showed that Cf-ID1 and Cf-ID2 were established in the nucleus and also in the cytoplasm.

## Discussion

In this study, UMI-RNA-seq technology was used to explore the infection strategy of *C. fructicola* in pecan. Many terms and pathways associated with the pathogenic process and candidate effectors were found, which provided strong evidence for revealing the pathogenic mechanism of *C. fructicola* in pecan.

Ribosomes are the main site for protein synthesis in eukaryotes, providing various proteins necessary for cell growth and development (Li et al., 2019). Multiple amino acids play important roles in the growth and proliferation of pathogenic fungi, among which, tyrosine is involved in the synthesis of melanin (Ito et al., 2000). Based on the infection process of *C. fructicola*, the ribosomes and ribosome biogenesis in eukaryote pathways were significantly enriched, and various amino acid metabolisms, such as alanine aspartate and glutamate metabolism, cysteine and methionine metabolism, and tyrosine metabolism, were also enriched at 6 hpi. Therefore, we speculated that at this early stage of infection of pecan, ribosome-related pathways might be related to spore germination and mycelial growth of *C. fructicola*, and various amino acid metabolisms might promote the maturation and melanization of appressoria, which was one of the infection structures of *C. fructicola*.

In the transcriptome analysis of *C. fructicola* infecting apple leaves, the response to oxidative stress was significantly enriched during the appressorium formation stage and the apple leaf infection stage (Liang et al., 2018). In this study, the ROS-related terms (response to oxidative stress, hydrogen peroxide catabolic process, oxylipin biosynthetic process, cellular oxidant detoxification, and cellular response to oxidative stress) and metabolic pathways (peroxisome and oxidative phosphorylation) were all significantly enriched at 6, 24, and 36 hpi, which suggested that oxidative stress from pecan might be one of the major obstacles to the successful infection by *C. fructicola* during the early infection stages. Correspondingly, *C. fructicola* also hydrolyzes the ROS produced by pecan to survive.

Previous studies have shown that fungal cytochrome P450 not only mediated the detoxification of exogenous toxic compounds, enabling them to adapt to harsh environments, but also participated in the biosynthesis of fungal ergosterol and toxins, which was beneficial for fungal infection of plant hosts (Črešnar and Petrič, 2011; Jawallapersand et al., 2014). Moreover, in the transcriptome of the early stages of migratory phytoparasitic nematode *Bursaphelenchus xylophilus* infection in pine, the drug metabolism cytochrome P450 pathway was also significantly enriched during the infection stage (Hu et al., 2021). GPI anchoring was a common post-translational modification in eukaryotic cells, and reports have shown that GPI anchor biosynthesis-related proteins were essential for the nutritional growth and pathogenesis of *Colletotrichum graminicola* (Mei et al., 2022). In plant pathogenic fungi, sphingolipids, their derivatives, and the MAPK signaling pathway have been proven to regulate various cellular processes, in which pathogens adapted to their hosts and played a crucial role in the regulation of fungal pathogenicity (Gong et al., 2017; Zhu et al., 2023). In this study, at 24 and 36 hpi, not only was the drug metabolism cytochrome P450 metabolic pathway significantly enriched, but metabolic pathways such as the MAPK signaling pathway, GPI-anchor biosynthesis, and sphingolipid metabolism were also significantly enriched. This indicated that after infecting pecan, *C. fructicola* initiated its own detoxification strategy and nutrient uptake to adapt to the host environment. GPI anchor biosynthesis-related proteins and sphingolipids and their derivatives participated in the pathogenic process of *C. fructicola*.

A primary study showed that potassium transporter CgTrk1 of *C. gloeosporioides* was involved in invasive growth and full virulence (Wang et al., 2024). Two-component and phosphorelay signal transduction systems of pathogenic bacteria control the expression of genes encoding virulence factors and essential functions (Stephenson and Hoch, 2002). For example, the virulence of *Pseudomonas syringae* was inhibited via phosphorelay crosstalk with sense polyphenols (Xie et al., 2021). In our study, potassium ion transport, phosphorelay signal transduction system, and exocytosis were significantly enriched during the early infection stages. This suggested that *C. fructicola* might secrete toxic substances (such as effectors and toxins) to promote infection through the abovementioned pathways. Moreover, protein ubiquitination and glycosylation have been shown to be closely related to the survival, reproduction, and virulence of pathogens, and some effectors can evade recognition by the host immune system through ubiquitination and glycosylation (Chen et al., 2017; Qiu and Luo, 2017). In this study, protein ubiquitination and glycosylation were significantly enriched in the early stages of infection, which indicated that protein post-translational modification was also one of the important infection strategies of *C. fructicola* in pecan.

At present, several important effectors have been screened and validated using the method of combining transcriptome sequencing with transient expression technology in *N. benthamiana* (Hu et al., 2019; Shang et al., 2020; Wen et al., 2021). For example, the effector CfEC92 was first screened from the transcriptome data in *C. fructicola* and it inhibited BAX-triggered cell necrosis in *N. benthamiana*. Indeed, the contribution of CfEC92 to pathogenicity was subsequently confirmed by gene knockout. Similarly, in this study, 191 candidate effectors were predicted, and two effectors were successfully characterized using the same method. This indicated that this screening method was effective. It was noteworthy that most candidate effectors were pioneer genes without any annotation, which was consistent with a previous study (Espada et al., 2016). The types of candidate effectors in our study included those predicted by previous researchers, such as pioneer genes, NLPs, small secreted proteins, and surface-binding proteins (Liang et al., 2018). Moreover, other candidate effectors, including allergens, some cell wall-degrading enzymes (CWDEs, such as pectate lyase), hydrolases (such as SL-like lipase and glycosyl hydrolases), and ribosomal proteins have been also identified as candidate effectors in *B. xylophilus* (Tsai et al., 2016; Hu et al., 2021). In a previous study, allergens were proven to target plant signaling pathways and suppress host defenses (Haegeman et al., 2012). CWDEs were important for breaking down plant cell walls to establish infection (Sonah et al., 2016; Qu et al., 2021). Several lipases of pathogenic bacteria and fungi have been identified as virulence factors (Stehr et al., 2003). Some ribosomal proteins participated in the interaction between *Meloidogyne incognita* and *Solanum lycopersicum* (Shukla et al., 2018). Thus, it was speculated that *C. fructicola* secreted these candidate effectors to promote colonization in pecan in different ways, such as degradation of the host cell wall, suppression of host defenses, or being toxic to host cells. In addition, the candidate effectors we screened provided an important database for further identifying key effectors during the interaction progress between *C. fructicola* and pecan.

In primary studies, multiple effectors that can trigger plant cell necrosis and trigger plant immunity have been identified in plant pathogens, for example, *Phytophthora sojae* effectors PsXEG1 and Ps238 (Ma et al., 2015; Yang et al., 2017) and *B. xylophilus* effectors BxSapB1 and BxSapB2 (Hu et al., 2019; Zhao et al., 2020). At present, Han et al. have identified four effector genes, CfCE4, CfCE25, CfCE61, and CfCE66, secreted by *C. fructicola* during the infection of pear trees, which can trigger cell death in *N. benthamiana* (Han et al., 2024). This study identified two effectors, Cf-ID1 and Cf-ID2, of *C. fructicola*, which not only induced cell death, but also triggered the immune and defense responses to suppress infection of *C. fructicola* in *N. benthamiana*, thus adding two new members to such effectors. In the future, Cf-ID1 and Cf-ID2 are expected to be used in the development of immune inducers of pecan against anthracnose, which will provide new ideas for the prevention and control of anthracnose of pecan. Research on the mechanism of plant immune responses triggered by the effectors Cf-ID1 and Cf-ID2 of *C. fructicola* by gene knock-out and the target protein of pecan will be the focus of future research. Moreover, we will also identify and characterize effectors secreted by *C. fructicola* that inhibit plant immunity when interacting with pecan and research their function mechanism.

## Data Availability

The datasets presented in this study can be found in online repositories. The names of the repository/repositories and accession number(s) can be found in the article/**Supplementary Material**.
