# Supplementary material for: Exploring the infection strategy of Colletotrichum fructicola in pecan and two effectors Cf-ID1 and Cf-ID2 were characterized using unique molecular identifier-RNA sequencing technology
Source: Front Plant Sci. 2025 Apr 17;16:1551342. doi: 10.3389/fpls.2025.1551342 (PMC12043710; doi:10.3389/fpls.2025.1551342)
Supplement: Supplementary file 1 [file DataSheet1.docx]

Supplementary Material

## Supplementary Figure S1


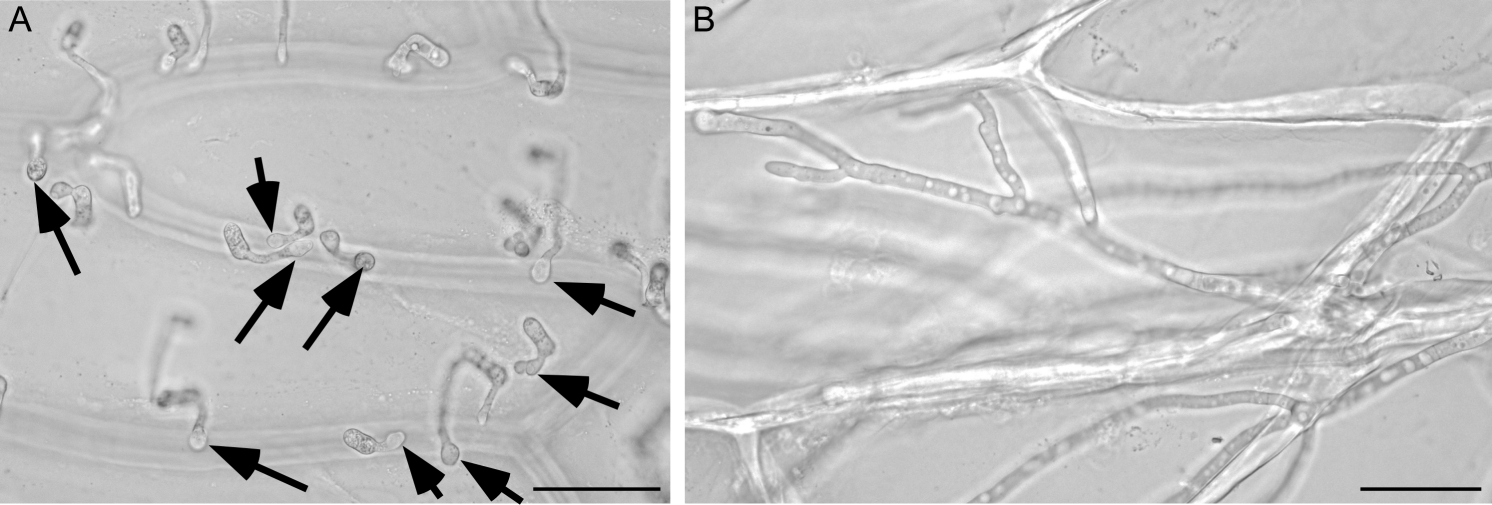


**Figure S1 Microscopic observation of infection structures formed by *Colletotrichum fructicola* in onion epidermal cells.** (A) Appressorium formation by *C. fructicola* conidia at 6 h post-inoculation (hpi). The black arrows point at the appressoria. (B) Primary hyphae formation by *C. fructicola* conidia at 24 hpi.

## Supplementary Figure S2


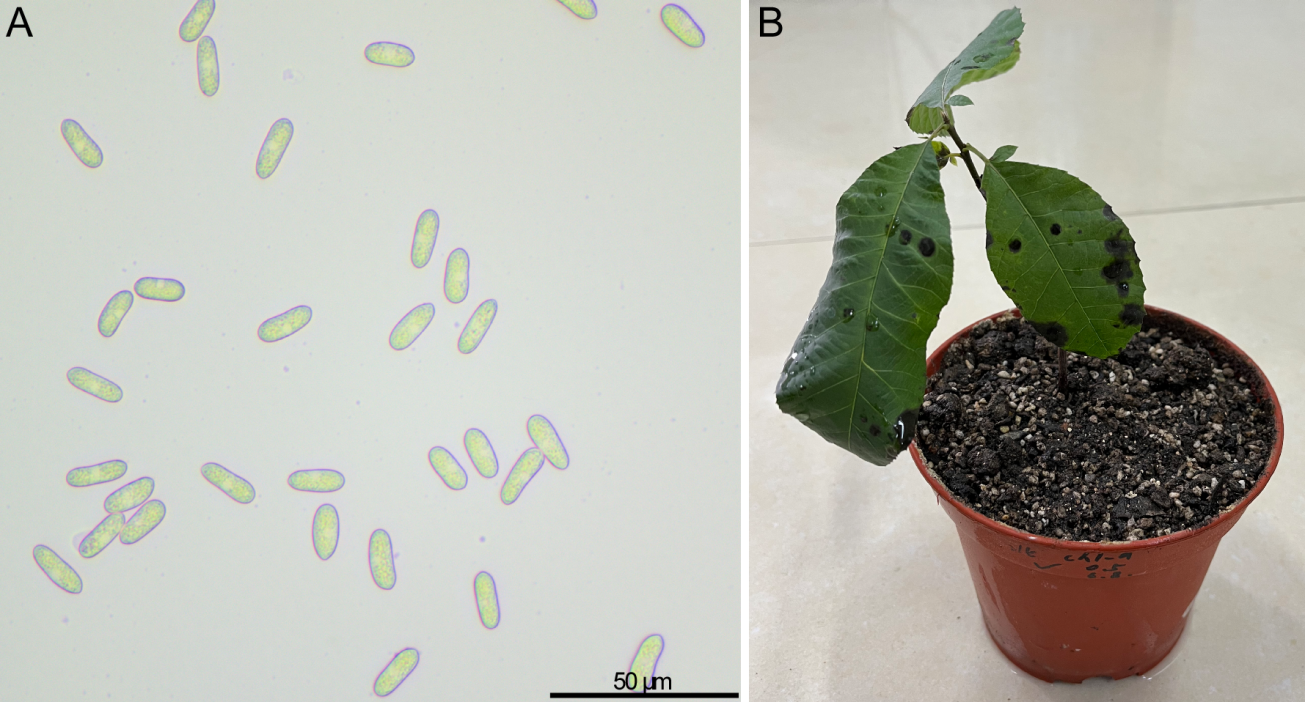


**Figure S2 Pathogenicity of *Colletotrichum fructicola* to pecan.** (A) Conidia morphology of *C. fructicola.* (B) Representative symptoms of pecan leaves at 3 days after inoculation with *C. fructicola*.

## Supplementary Figure S3

**Figure S3 The heatmap of differential expressed genes (DEGs) of *Colletotrichum fructicola* at the early infection stages in pecan.**

## Supplementary Figure S4


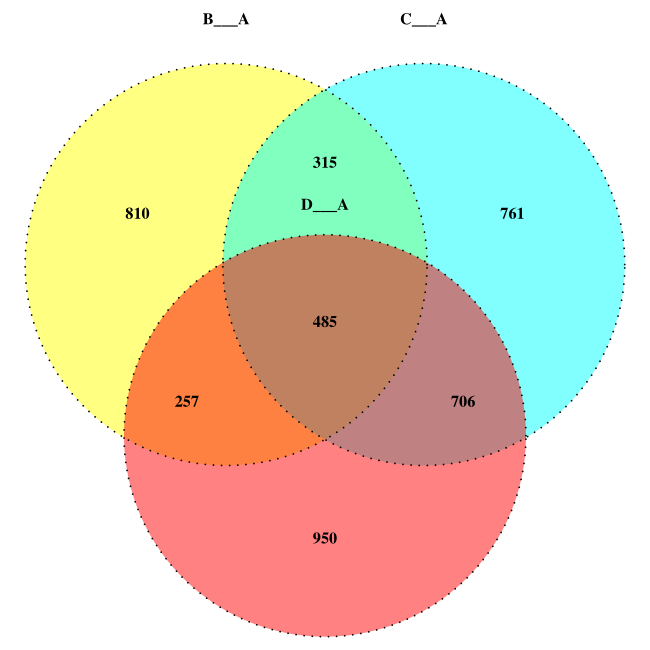


**Figure S4** **The Venn diagram analysis of upregulated genes of *Colletotrichum fructicola* at the early infection stages.**

## Supplementary Figure S5


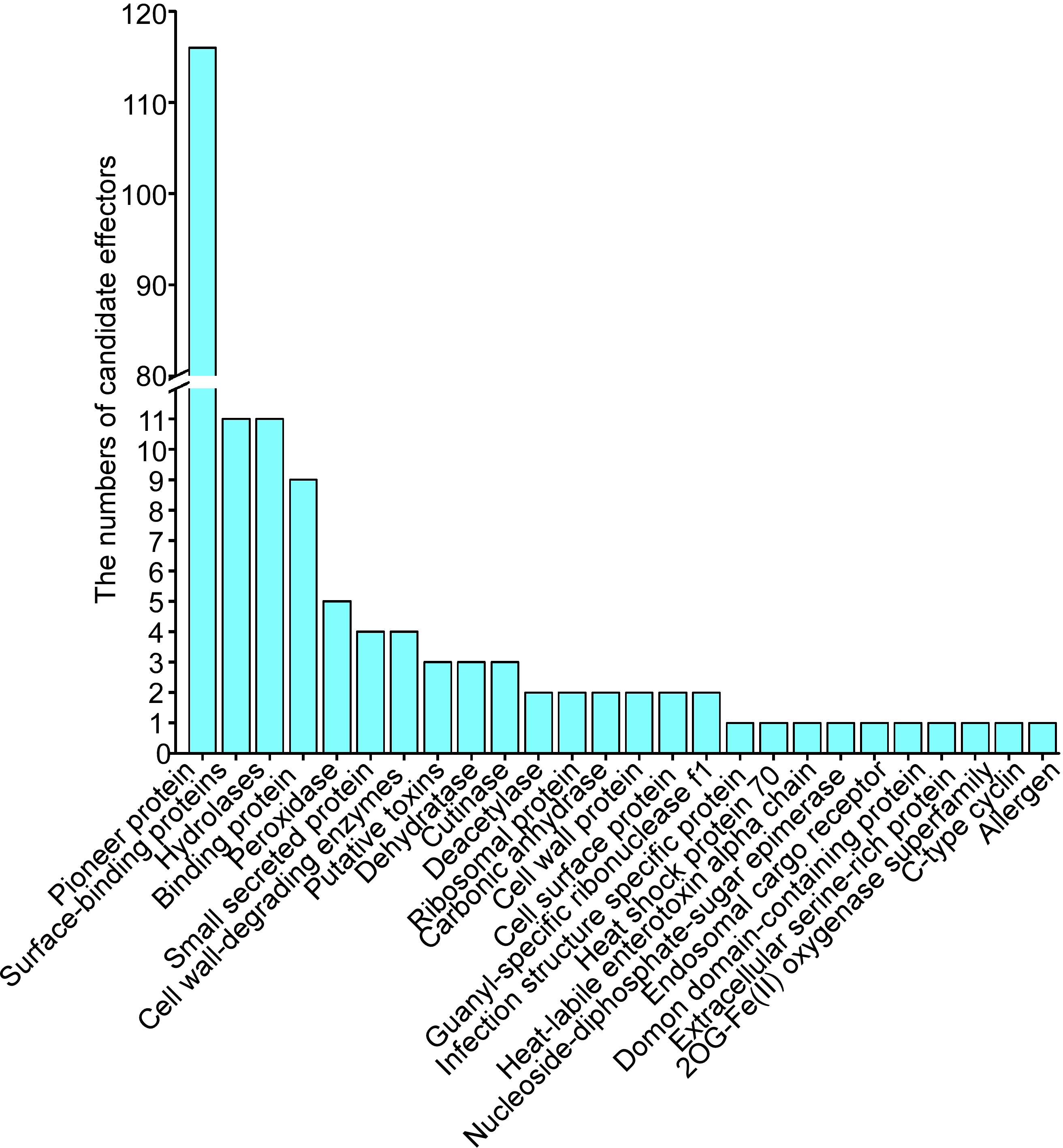


**Figure S5** **The annotation information of 191 candidate effectors in *Colletotrichum fructicola.***
